# Integration of Light and Auxin Signaling in Shade Plants: From Mechanisms to Opportunities in Urban Agriculture

**DOI:** 10.3390/ijms23073422

**Published:** 2022-03-22

**Authors:** Xiulan Xie, Hao Cheng, Chenyang Hou, Maozhi Ren

**Affiliations:** 1 Laboratory of Space Biology, Institute of Urban Agriculture, Chinese Academy of Agricultural Sciences, Chengdu 610213, China; xiexiulan01@caas.cn (X.X.); chenghao01@caas.cn (H.C.); 2 Zhengzhou Research Base, State Key Laboratory of Cotton Biology, School of Agricultural Science of Zhengzhou University, Zhengzhou 450000, China; h937998560@gmail.com; 3 Hainan Yazhou Bay Seed Laboratory, Sanya 572025, China

**Keywords:** avoidance, photomorphogenesis, suboptimal light, tolerance, vertical planting

## Abstract

With intensification of urbanization throughout the world, food security is being threatened by the population surge, frequent occurrence of extreme climate events, limited area of available cultivated land, insufficient utilization of urban space, and other factors. Determining the means by which high-yielding and high-quality crops can be produced in a limited space is an urgent priority for plant scientists. Dense planting, vertical production, and indoor cultivation are effective ways to make full use of space and improve the crop yield. The results of physiological and molecular analyses of the model plant species *Arabidopsis thaliana* have shown that the plant response to shade is the key to regulating the plant response to changes in light intensity and quality by integrating light and auxin signals. In this study, we have summarized the major molecular mechanisms of shade avoidance and shade tolerance in plants. In addition, the biotechnological strategies of enhancing plant shade tolerance are discussed. More importantly, cultivating crop varieties with strong shade tolerance could provide effective strategies for dense planting, vertical production, and indoor cultivation in urban agriculture in the future.

## 1. Introduction

**Figure 1:**
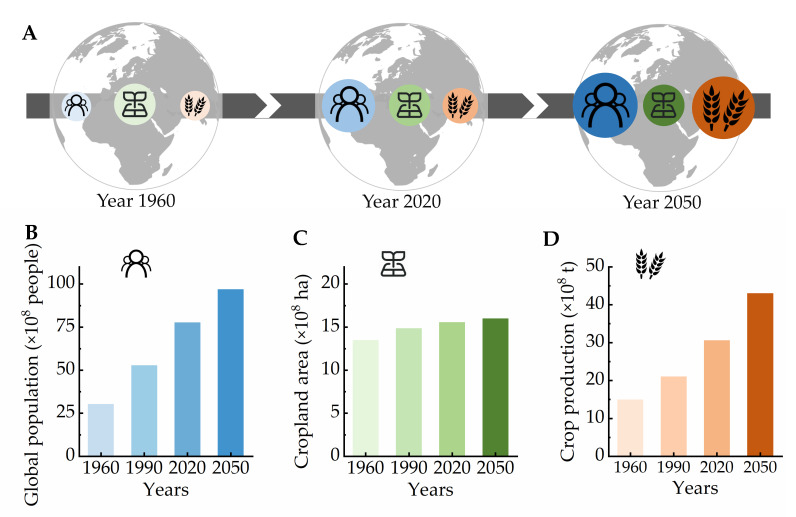
Variation in the global population, cropland area, and crop production from 1960 to 2050. (**A**) Past (1960), present (2020), and future (2050) estimates of global population, cropland area, and crop production. With the world population continuously growing and cropland area remaining largely unchanged, crop production must increase to fulfill the basic needs of the global population. Making full use of limited space is an effective way to improve the crop yield. (**B**–**D**) Estimated global population, global cropland area, and global crop production for the 1960–2050 period. To fully meet the needs of 10 billion people, crop production should increase by 50% on the available land by 2050. The global population data were obtained from the World Bank (https://databank.worldbank.org/home.aspx, accessed on 7 January 2022); the cropland area and crop production data were downloaded from the FAO website (https://www.fao.org/faostat/en/#data, accessed on 7 January 2022).

In the context of rapid population growth, frequent extreme climate events, and limited arable cultivated lands, traditional rural agriculture is facing unprecedented challenges (Figure 1A). According to the Food and Agriculture Organization of the United Nations (FAO) statistics, the global population increased from 5 billion in 1990 to 7.5 billion in 2020 and is expected to increase to nearly 10 billion by 2050 (Figure 1B). It is estimated that the cropland area will remain at approximately 1.5 billion hectares from 1990 to 2050 (Figure 1C). To fully meet the food needs of 10 billion people, crop production on the available arable land needs to increase by 50% by 2050 (Figure 1D) [1]. In addition, according to the World Bank, the world urbanization rate increased from 33.62% in 1960 to 56.16% in 2020. Amid this intensification of urbanization, problems of urban construction, population, and industrial density are prominent, leading to significant urban heat island effects and serious air pollution [2]. However, cities worldwide have more than 300 million mu (a Chinese unit of area, equal to 1/15 of a hectare) of roof, balcony, indoor space, and other available space and human resources of >700 million urban elderly (aged ≥65 years) who do not have disabilities (https://databank.worldbank.org/home.aspx, accessed on 7 January 2022). Furthermore, there is an urgent demand for urban agriculture with ecological, habitation, and food production functions.

Dealing with the food challenge that will be brought about by the future population surge and improving the yield, quality, and stress resistance of crops cultivated in a limited space are pertinent issues. Dense planting, vertical production, and indoor cultivation are effective ways to make full use of space and improve the crop yield. However, the low-light environment that these cultivation methods face affects important agronomic traits, such as plant type, plant height, stress resistance, and growth period, and it reduces crop quality and yield. This is because light is one of the most important environmental factors affecting crop growth, development, flowering, and fruit bearing [3]. Fortunately, as a result of evolution, domestication, and improvement, many plants have developed coping strategies to deal with shading or weak light, that is, shade avoidance or shade tolerance.

Focusing on the auxin-mediated shade avoidance or shade tolerance of plants in response to changes in light quality and intensity, this paper presents a review of the physiological and biochemical characteristics of plants under different growth conditions. In addition, the major molecular mechanisms of shade avoidance and tolerance are summarized. Furthermore, in this paper, we propose the biotechnological strategies for enhancing plant shade tolerance. Cultivating crop varieties with strong shade tolerance could provide effective strategies for dense planting, vertical production, and indoor cultivation of urban agriculture in the future.

## 2. Shade Avoidance and Tolerance in Plants

**Figure 2:**
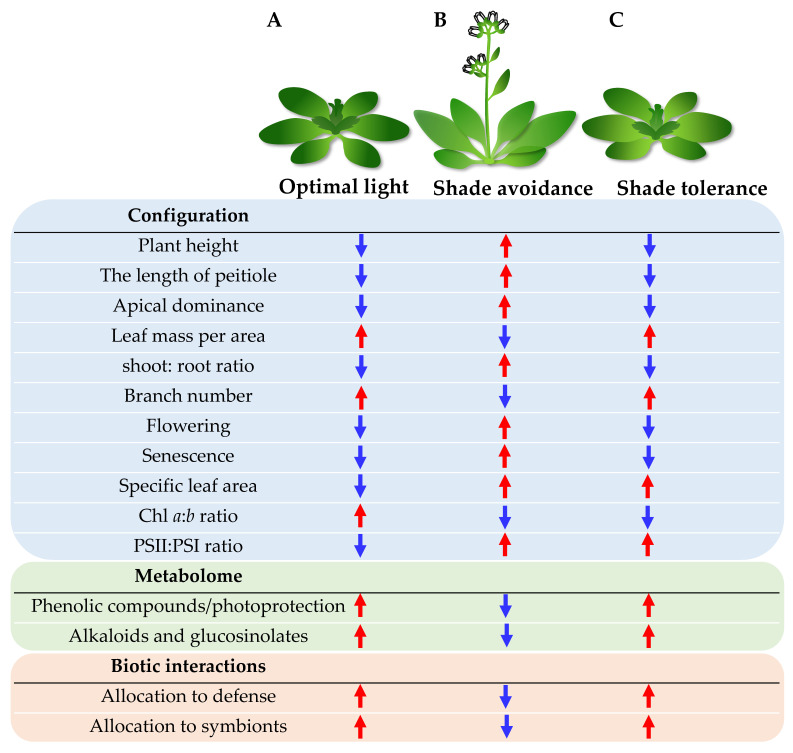
Physiological and biochemical responses of plants to optimal light, and suboptimal light (as in shade avoidance and shade tolerance mechanisms). (**A**) Changes in plant configuration, metabolites, and biotic interactions under optimal light conditions. (**B**) Changes in configuration, metabolites, and biotic interactions of shade avoiders under shading and suboptimal light. (**C**) Changes in configuration, metabolites, and biotic interactions of shade-tolerant species to shading and suboptimal light. Red arrows denote increasing trend, and blue arrows denote decreasing trend. See main text for details. Abbreviations: Chl, chlorophyll; PS, photosystem.

Photosynthetic plants have evolved a series of complex light responsive mechanisms to adapt to and utilize the light environment, thus accurately regulating their growth and development. Light signals in the environment are first perceived by specific light receptors in plants. After receiving the light signal from the environment, plant photoreceptors transmit and amplify this signal step-wise, trigger plant physiological and biochemical responses, and mediate plant morphogenesis [4]. Different light conditions cause significant differences in plant morphological structure, metabolism, and interaction with other organisms (Figure 2) [5]. Under sufficient light conditions, short phenotypes with well-photoprotected and thick leaves are generally favored, because this configuration maximizes light capture and utilization in an open habitat. At the same time, short stature minimizes damage from wind and aboveground herbivores [6]. In addition, the developed plant roots under sufficient light are able to meet a higher water demand and increase the utilization of mineral nutrients (Figure 2A) [7].

Under the conditions of dense planting and vegetation canopy, there is a decrease in the intensity of the light reaching the plants, the ratio of red light: far-red light (R:FR), and the activity of photoreceptors [8]. Furthermore, plants coordinate and control their growth and development, such as plant height, branch number, flowering, senescence, and disease resistance, through integration with hormone pathways [9]. Research has shown that shade-intolerant plants stimulate a series of shade-avoidance-related responses (SARs) (Figure 2B), which leads to changes or specializations in leaf morphology, chloroplast structure, photosynthetic pigment content, light quantum yield, and other parameters. This ensures that light energy can continue to be fully and efficiently utilized under low-light conditions and that the energy balance required for growth is maintained. Changes in morphological structure, including increased plant height, petiole elongation, and evident apical dominance, could help plants to avoid canopy cover and increase the specific leaf area to improve the ability of light energy interception, allow for early flowering and senescence, complete reproduction as soon as possible, and cause a sharp reduction in branch numbers (tillers) [9]. Light intensity also has a direct impact on pigment formation, content, and distribution, and the pigment–protein complexes on the chloroplast photosynthetic membrane [10]. Weak light increases the Chl *b* content, decreases the Chl *a*:Chl *b* ratio, increases the light-harvesting pigment complex content, and increases the photosystem-II(PSII)-to-photosystem-I (PSI) ratio [11]. In addition to morphological changes, sun–shade transformation also leads to a large number of metabolite adjustments [12]. Defense-related metabolites, such as phenolic compounds and alkaloids, are usually downregulated under shade, whereas volatile terpenes display uncertain changes [13]. This downregulation of chemical defense and morphological changes caused by shade avoidance usually makes plants more vulnerable to attacks by other organisms, which reduces the symbiotic associations between the plants and microorganisms [5]. Eventually, shade-intolerant plants produce a slender-plant phenotype under low light to maximize their SAR.

Compared to plants with weak shade tolerance, some plants with strong shade tolerance do not respond significantly to the shade avoidance mentioned above. They can adapt to light changes only through partial physiological and metabolic changes; however, this is usually accompanied by a lower growth rate. This process is referred to as shade-tolerance-related response (STR) [14]. The physiological changes triggered by STR include a decreased Chl *a*:Chl *b* ratio and an increased PSII:PSI ratio (Figure 2C). In general, shade-tolerant plants have a stronger ability to adapt to low-light environments.

## 3. Mechanism of Light and Auxin Integration in SAR and STR

**Figure 3:**
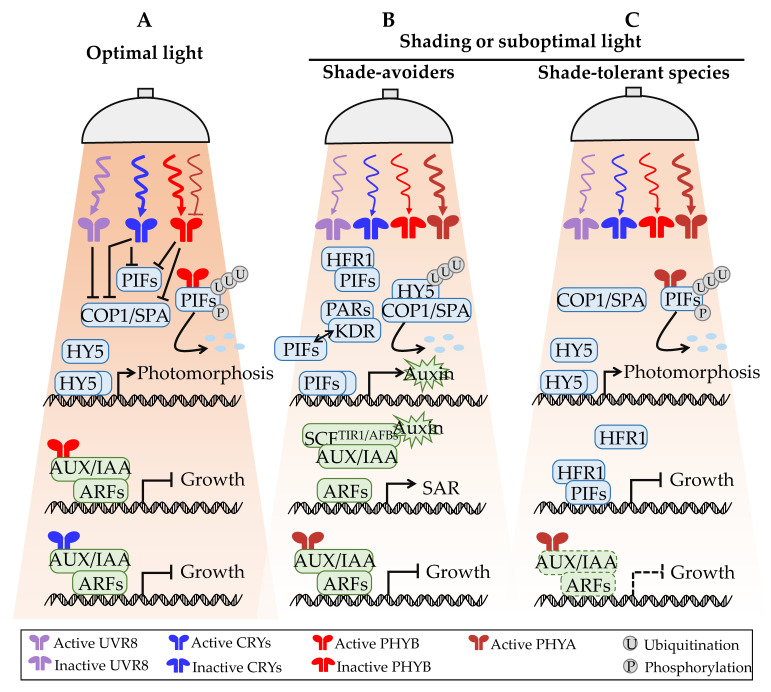
Mechanism of light and auxin integration in response to optimal light, shading, or suboptimal light in plants. (**A**) Photomorphogenesis of plants under optimal light distribution. (**B**) Shade-avoidance-related response (SAR) and the feedback mechanism of shade avoiders under shading or suboptimal light. (**C**) Shade-tolerance-related response (STR) mechanism of shade-tolerant species to shading or suboptimal light. Arrows indicate positive regulation, T-shaped lines indicate negative regulation. Dotted line indicates possible regulation. See main text for details. Abbreviations: PIFs, phytochrome interacting factors; COP1/SPA, constitutively photomorphogenic 1/suppressor of phytochrome A; HY5, elongated hypocotyl 5; HFR1, long hypocotyl in far-red 1; PARs, photosynthetically active radiation; KDR, KIDARI; AUX/IAA, auxin/indole-3-acetic acid; ARFs, auxin response factors; SCF, S-phase kinase-associated protein 1 (SKP1)-cullin 1 (CUL1)-F-box protein; TIR1/AFBs, transport inhibitor response 1/auxin signaling F-box proteins; UVR8, ultraviolet radiation 8; CRYs, cryptochromes; PHYA, phytochrome A; PHYB, phytochrome B; SAR, shade-avoidance-related response.

The differences in plant structure, physiology, and biochemistry under different light conditions (see Figure 2) are attributed to the step-wise transmission of light signals and the coordinated control of endogenous hormones, which trigger downstream key transcription factors to regulate the expression of genes related to plant development. Extreme shade-tolerant species exist in all plant types, from simple to complex, nonvascular to vascular, and aquatic to terrestrial, and include marine plants, mosses, ferns, gymnosperms, and angiosperms. Previously, this subject was thoroughly investigated in the model plant *Arabidopsis thaliana*. Hence, taking *A. thaliana* as the model and combining the research results for other species, this section focuses on the photomorphogenesis of plants, the SAR triggered by shading of shade-intolerant plants, the STR core pathway of shade-tolerant plants, and the cross-talk of auxin in the STR pathway under optimal light distribution (Figure 3).

### 3.1. Photomorphogenesis of Plants under Optimal Light Distribution

Under sufficient light conditions, photoreceptor proteins, such as ultraviolet radiation 8 (UVR8), cryptochromes (CRYs), and phytochromes (PHYs), are activated after receiving light signals and combine with constitutively photomorphogenic 1 (COP1) to inhibit the nuclear localization of plant hypocotyl COP1 (Figure 3A). By remaining in the cytoplasm, COP1 cannot accelerate the hydrolysis of elongated hypocotyl 5 (HY5) in the nucleus. Therefore, HY5 promotes the expression of genes related to photomorphogenesis, thus inhibiting the elongation of the hypocotyl [15]. In addition, the suppressor of phytochrome A 1 (SPA1) can form a stable heterodimer with COP1, thereby enhancing the E3 ubiquitin ligase activity of COP1 [16]. However, most phytochrome-interacting factors (PIFs) (such as PIF1/3/4/5) are rapidly phosphorylated after interacting with activated photoreceptors PHYs and CRYs and are then degraded by proteasome through ubiquitination [17]. In contrast, UVR8 cannot directly interact with PIFs [18]. The research results show that PIF5 interacts with COP1/SPAs to promote the stability of PIF5 in plants, whereas the combination of UVR8 and COP1/SPAs destroys this stability, providing a mechanism for UV-B to rapidly reduce the PIF abundance under light conditions [19]. Furthermore, UVR8 binds to WRKY36 in the nucleus and inhibits its DNA-binding domain activity, thereby enhancing the transcriptional activity of HY5. The UVR8-WRKY36-HY5 is another newly discovered mechanism of plant photomorphogenesis mediated by the UV-B signal transduction pathway [20].

In addition to the direct regulation of the light signal pathway, plant photomorphogenesis also proactively regulates plant growth by establishing contact with the plant hormone system [21]. At the hormone level, light produces a strong impact on many aspects of the auxin system, controlling the auxin level, its transportation, and response ability. The auxin signaling pathway has been described in detail [22,23,24] and is not repeated in this paper. Auxin/indole-3-acetic acid (AUX/IAA) protein stability is also controlled by light [25]. Under high-intensity red or blue light, PHYB or CRY1 interacts with AUX/IAA protein in a light-dependent manner, thereby inhibiting AUX/IAA protein degradation and inhibiting the transcriptional regulation function of auxin response factors (ARFs), and ultimately reducing hypocotyl elongation [26]. In addition, PHYB and CRY1 can also interact directly with ARF proteins, such as ARF6 and ARF8, to inhibit their DNA binding ability [27]. These studies revealed that AUX/IAA stability is rapidly regulated by auxin receptors and photoreceptors. In this way, plants can sense the endogenous auxin content and ambient light signals to fine-tune cell elongation under different light conditions.

### 3.2. SAR and the Feedback Mechanism of Shade Avoiders under Shading or Suboptimal Light

Early studies on plant responses to shading mainly focused on the verification of SAR-related gene functions [28,29]. Many studies have shown that PIFs are positive regulators of SAR. Transgenic experiments prove that the elongation growth of single-mutant *pif4* and *pif5* and double-mutant *pif4pif5* was slower than that of the wild type, and that overexpression of *pif5* in plants can lead to constitutive SAR even without shading [30,31,32]. Similarly, PIF1 and PIF3 also participate in SAR; however, their contribution is weaker than that of PIF4 and PIF5 [33]. *A. thaliana* plants overexpressing *PIF1*, *PIF3*, *PIF4*, and *PIF5* showed SAR even under normal light, whereas *pifq* (*pif1pif3pif4pif5*) mutants produced short petioles and weakened responses to shading [34]. Compared with *PIFq* (*PIF1*, *PIF3*, *PIF4*, and *PIF5*) members, *PIF7* played a leading role in PHYB-mediated SAR because *pif7* mutants had a more severe shade-defective phenotype [35,36]. The residual response of *pif7* and *pifq* mutants to a low R:FR ratio indicates that there are other unknown pathways or factors controlling this process [36]. In addition to PIFs, a shadow environment can upregulate auxin synthesis, which is another core component of SAR [37]. Interruption of auxin transport in *pin3* and *sav4* mutants can affect shadow-induced hypocotyl elongation, emphasizing the importance of auxin transport in SAR [38]. In addition, many auxin signal components, such as IAA17, IAA19, IAA29, and GH3.3, are also involved in SAR [39]. Furthermore, ARF family members, ARF6, ARF7, and ARF8, have been shown to be necessary for auxin-mediated hypocotyl elongation [40]. In summary, the functional verification of core genes in the SAR pathway enhances the understanding of the SAR mechanism.

In recent years, scientists have made great progress in understanding the mechanism of plant-mediated SAR. As a shade-avoiding model plant, *A. thaliana* provides a foundation for the study of genetic and molecular mechanisms of SAR regulation (Figure 3B). The results show that under shadow conditions, the proportion of active PHYB and CRYs decreased or existed in the cytoplasm in an inactive form, thereby increasing the rapid accumulation of PIF proteins in the nucleus [41]. On the one hand, these PIF proteins can directly bind to the promoters of multiple members of the miR156 gene families and inhibit the expression of the miR156 genes, resulting in the increased expression of target SQUAMOSA-promoter binding-like (SPL) gene family members [42]. SPL genes are targeted by miR156 family members and further regulate a series of critical agronomic traits, such as plant height, branch number, petiole length, number of leaves, leaf area, and the time of flowering [43,44,45]. On the other hand, deubiquitination of PIF proteins and their stable accumulation in plant cells [46] can promote the expression of auxin synthesis genes and the rapid accumulation and transport of IAA [47,48,49,50]. For example, PIF7 can directly activate downstream target genes, including auxin biosynthesis genes *YUCCA8* and *YUCCA9*, at a low R:FR ratio [51]. The polar transport of the synthesized auxin from the cotyledon to the hypocotyl and from the internal tissue to the epidermis is regulated by auxin transporters PIN3, PIN4, and PIN7 [52,53]. At increased concentrations of free auxin, the auxin receptor transport inhibitor response 1/auxin signaling F-box proteins (TIR1/AFBs) binds auxin, which enhances the interaction between TIR1/AFBs and AUX/IAA proteins and leads to ubiquitination of the latter. AUX/IAA protein is degraded by 26S proteasome to release ARF, which itself forms a homodimer to promote the transcription of SAR-related genes [54]. COP1 is located in the nucleus and interacts with HY5. SPA1 enhances the E3 ubiquitin ligase activity of COP1, leading to the ubiquitination of HY5 and degradation by the 26S proteasome. Subsequently, SAR is initiated, resulting in hypocotyl elongation. In addition, inactive PHYB and CRYs in the cytoplasm cannot physically interact with AUX/IAA protein [26]. Similarly, another study on *Nicotiana attenuata* revealed that NaZTL (a circadian clock component and blue-light photoreceptor) plays a role in SAR by directly interacting with NaPHYB and regulating the transcriptional levels of *PHYBs*, *PIF3a*, *PIF7*, and *PIF8* under shading conditions [55].

In the case of severe shading, plants also evolve a series of feedback mechanisms to inhibit SAR (Figure 3B). The stability of PHYA protein is significantly enhanced under shading conditions. The interaction between PHYA and AUX/IAA affects the interaction between auxin receptor TIR1 and AUX/IAA, thus preventing the degradation of AUX/IAA mediated by TIR1 [56]. Consequently, the auxin signal is weakened, which inhibits the excessive growth caused by long-term exposure to low light. In addition, PIF activity also plays an important role in the feedback loop. PIFs activity is regulated by PIF-interacting proteins, including long hypocotyl in far-red 1 (HFR1), photosynthetically active radiation 1 (PAR1), and PAR2 [57]. These proteins do not directly bind to DNA but inhibit the binding of PIFs to target sequences on DNA after binding to PIF proteins, thereby inhibiting the activity of PIF transcription factors [58]. In contrast, bHLH48/bHLH60, as the positive protein partner of PIF7, enhances the DNA-binding ability of PIF7 and promotes hypocotyl elongation after binding to PIF7 [59]. Furthermore, recent research results show that PHYA facilitates the expression of the circadian clock components TIMING OF CAB EXPRESSION 1, PSEUDO RESPONSE REGULATOR 7, EARLY FLOWER 3, and EARLY FLOWER 4, and that these component proteins are directly target-bound to the PIF promoter to increase the inhibition of PIF activity [60]. Thus, stem elongation is jointly inhibited to resist SAR in deep shade [61]. In short, bHLH transcription factors form a tertiary regulatory network to regulate the SAR of plants, in which KIDARI (KDR1) binds to PARs and competitively inhibits the binding ability of PIFs to PARs, thereby releasing PIF proteins and activating downstream events [62].

In conclusion, the discovery of the positive regulation pathways of PIFs-auxin and PHYs-PIFs-miR156-SPLs and the negative feedback signals of HFR1, PARs, and PHYA-AUX/IAA-ARFs have greatly improved our understanding of the regulation mechanism of SAR and laid a theoretical foundation for the cultivation of new varieties of shade-tolerant and densely planted crops.

### 3.3. STR Mechanism of Shade-Tolerant Species in Shading or Suboptimal Light

Approximately 450,000–500,000 plant species have adapted to different living environments, and some of those species thrive in low-light environments [63]. These plants inhabit dense forests, dark and humid mountain streams, and deep oceans or are grown indoors and on balconies. More importantly, these shade-tolerant plants provide valuable germplasm resources to accommodate the needs of dense planting and urban agriculture.

Limited research progress has been made in this field with previous studies mainly focusing on the phenotypic and basic physiological analysis of crops, such as those used in horticulture [64,65], and forest plants [66]. For example, Morelli et al. [67] determined *A. thaliana* to be a shade-avoiding species and *Cardamine hirsute* to be a shade-tolerant species. The chlorophyll and carotenoid levels decreased by approximately 20% in *A. thaliana* plants grown under low-R:FR conditions, whereas the decrease in chlorophyll and carotenoids was attenuated in *C. hirsuta* plants [68]. In addition, the green algae *Koliella antarctica* in polar ecosystems (Antarctica and Arctic) can carry out photosynthesis under low-light intensity in extreme environments. A study of its physiological and morphological response showed that *K. antarctica* has lutein epoxide and lutein, which may be essential for its normal growth under very low light intensity (<10 µmol m^−2^  s^−1^) or even prolonged darkness (>60 d) [69]. In general, most researchers studying STR in different species have attempted to use the method of comparative study to explain the possible STR mechanism of shade-tolerant plants.

In comparison, a gap remains in the current research on the STR mechanism. As a shade-avoiding plant, *A. thaliana* has been the subject of many thorough studies on the SAR mechanism. Nevertheless, *A. thaliana* is not suitable for studying the shade tolerance mechanism. Fortunately, its close relative *Cardamine hirsuta* usually grows in the shade, and its hypocotyl elongation is not a response to suboptimal light in the shade avoidance mechanism [68]. Instead, *C. hirsuta* survives through shade tolerance. Genetical screening of *C. hirsuta* lines with long hypocotyls in the ethyl methanesulfonate mutant population revealed that the *sis1* mutant lines lacked PHYA, whereas the *phyA*-deficient *A. thaliana* seedlings showed a response pattern similar to that of *sis1* seedlings [68]. This indicates that the negative tolerance of *C. hirsuta* may be due to the existence of a PHYA-dependent inhibition mechanism, which inhibits hypocotyl elongation and other SARs. The function of PHYA was not restored after supplementing the *phyA* mutant of *A. thaliana* with PHYA of *C. hirsute*, indicating that the phytochrome from these two different sources had changed, but they were functionally nonexchangeable, with PHYA in *C. hirsuta* having a higher expression level and activity than that in *A. thaliana* [68]. Additionally, the enhancement of PHYA activity is not sufficient to fully explain the STR of *C. hirsuta* in the shade. Recent studies have found that the transcription regulator HFR1 plays an important role in this reaction. Specifically, HFR1 protein in *C. hirsuta* is more stable than its counterpart in *A. thaliana*, which most likely enhances the biological activity of HFR1 due to its lower binding affinity with COP1 [70]. The enhanced total HFR1 activity was accompanied by a decrease in PIF7 activity and weakened SARs that were mediated by other PIFs [70] (Figure 3C). Molecular models of PHYA and PIF7-HFR1, involving *C. hirsuta* as the model plant, were able to clarify some shade tolerance mechanisms of plants and provide ideas for future studies on other shade-tolerant species in terms of their adaptation principles.

## 4. Prospects: From Shade Avoidance and Shade Tolerance Mechanisms to Development of Shade-Tolerant Plants for Urban Agriculture

As previously mentioned, research on shade avoidance and shade tolerance mechanisms has mainly focused on *A. thaliana* and *C. hirsuta*. Despite the currently limited understanding of the SAR and STR mechanism, great progress has been made in recent years, and it is possible to isolate the genes related to shade avoidance and shade tolerance to manage the low R:FR ratio or low PAR. For example, the *Zmpif* mutation produced by knockout of a *ZmPIF* gene by CRISPR/Cas9 technology in maize severely inhibited the elongation of hypocotyl under dark growth conditions. Moreover, the *Zmpif* mutation can attenuate plant elongation in response to simulated shading treatment, thereby inhibiting excessive plant growth under shading, and ultimately reducing the plant height [71]. Similar to the expression of *PHYA* in *C. hirsuta*, several studies have indicated that modulating the expression of the oat *PHYA* gene can improve crop cultivars [72]. The improved traits include enhanced yield, improved turf quality, retarded plant height, and enhanced shade tolerance. The oat *PHYA* gene and its hyperactive mutant (*S599A-PHYA* [73]) have been functionally verified in selected crops, such as zoysiagrass (*Zoysia japonica*) [73], creeping bentgrass (*Agrostis stolonifera*) [73], rice (*Oryza sativa*) [74], potato (*Solanum tuberosum*) [75], tomato (*Lycopersicon esculentum*) [76], sweet potato (*Ipomea batatas*) [77], and *A. thaliana* [77]. The above comprehensive studies on cereals, horticultural plants, and turf plants confirm that the basic understanding of shade tolerance mechanism obtained from *A. thaliana* and oat can be potentially applied to other distant species.

With increasing urbanization globally, the agricultural form has gradually expanded from rural agriculture to urban agriculture [78]. This agricultural form is mainly distributed in cities, urban suburbs, and urban radiation economic circles. It aims to make full use of roofs, balconies, courtyards, indoor spaces, suburban areas, and other fragmented free spaces to develop and plant grains, cotton, oil, vegetables, fruits, medicinal plants, flowers, woody plants, and grasses. Urban agriculture is an efficient and high-quality agricultural model that integrates food production, urbanization, and ecology. It is of great significance for alleviating the urban heat island effect, purifying air quality, inheriting agricultural culture, alleviating stress and improving mental health, and improving the ability to respond to major diseases. In the future, understanding the means by which high-quality and high-yielding crops can be produced in a limited space and under low-light conditions is an urgent challenge for breeders and plant scientists. Although vertical farming is emerging as a new indoor planting method in various countries, its disadvantages of high energy consumption and high carbon emission prevent its popularization [79]. Direct use of living and working lighting to carry out indoor cultivation of crops is the future development trend and direction of urban agriculture.

**Figure 4:**
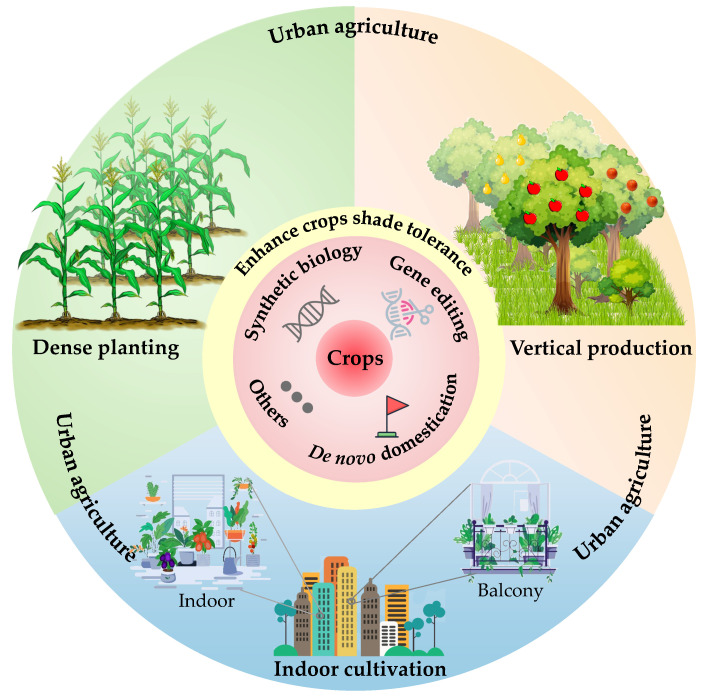
Application prospect of shade-tolerant plants in urban agriculture. To enable human society to keep pace with environmental change and population growth, more food needs to be produced in a limited space and under low-light conditions. Biotechnologies, such as gene editing, synthetic biology, and de novo domestication, help to accelerate the enhancement of crop shade tolerance, so as to adapt to dense planting, vertical production, and indoor cultivation, and their application to urban agriculture.

Effective methods that can contribute to solving these problems include mining of functional genes in shade-tolerant plants [77], expanding the spectral range of photosynthesis [80], designing light-harvesting antenna protein complexes adaptive to low-light conditions [81,82,83], and reducing the energy consumption of photorespiration [84,85]. The upper 200 m of the oceans, known as the “euphotic zone,” is the main gathering area of marine photosynthetic organisms. Marine photosynthetic organisms have evolved a strong and unique ability to adapt to low-light environments and can thrive even in the presence of weak light [86]. Further exploration of the adaption mechanism of marine photosynthetic oxygen-releasing organisms to low-light environments could provide a reference for cultivating shade-tolerant plants. In general, current biotechnology is sufficient to cope with the problem of limited light in urban agricultural crop cultivation (Figure 4). The combination of genotyping, marker-assisted screening, high-throughput phenotypic analysis [87], gene editing [88], genomic selection [89], synthetic biology [90], de novo domestication [91], rapid breeding [92,93,94], and the Whole-Body Edible and Elite Plants strategy [95] could potentially accelerate gene mining, trait analysis, and predictive breeding. This approach allows human society to keep pace with environmental changes and population growth.

## Data Availability

Not applicable.
